# Preliminary dosimetric study on feasibility of multi-beam boron neutron capture therapy in patients with diffuse intrinsic pontine glioma without craniotomy

**DOI:** 10.1371/journal.pone.0180461

**Published:** 2017-06-29

**Authors:** Jia-Cheng Lee, Keh-Shih Chuang, Yi-Wei Chen, Fang-Yuh Hsu, Fong-In Chou, Sang-Hue Yen, Yuan-Hung Wu

**Affiliations:** 1 Department Oncology, Taipei Veterans General Hospital, Taipei, Taiwan; 2 Department of Biomedical Engineering and Environmental Sciences, National Tsing Hua University, Hsinchu, Taiwan; 3 School of Medicine, National Yang-Ming University, Taipei, Taiwan; 4 Nuclear Science and Technology Development Center, National Tsing Hua University, Hsinchu, Taiwan; 5 Institue of Public Health, National Yang-Ming University, Taipei, Taiwan; 6 Department of Biomedical Imaging and Radiological Sciences, National Yang-Ming University, Taipei, Taiwan; National Cancer Institute, UNITED STATES

## Abstract

Diffuse intrinsic pontine glioma is a very frustrating disease. Since the tumor infiltrates the brain stem, surgical removal is often impossible. For conventional radiotherapy, the dose constraint of the brain stem impedes attempts at further dose escalation. Boron neutron capture therapy (BNCT), a targeted radiotherapy, carries the potential to selectively irradiate tumors with an adequate dose while sparing adjacent normal tissue. In this study, 12 consecutive patients treated with conventional radiotherapy in our institute were reviewed to evaluate the feasibility of BNCT. NCTPlan Ver. 1.1.44 was used for dose calculations. Compared with two and three fields, the average maximal dose to the normal brain may be lowered to 7.35 ± 0.72 Gy-Eq by four-field irradiation. The mean ratio of minimal dose to clinical target volume and maximal dose to normal tissue was 2.41 ± 0.26 by four-field irradiation. A therapeutic benefit may be expected with multi-field boron neutron capture therapy to treat diffuse intrinsic pontine glioma without craniotomy, while the maximal dose to the normal brain would be minimized by using the four-field setting.

## Introduction

Diffuse intrinsic pontine glioma (DIPG) is a very frustrating disease for patients, families, and doctors. Because of the tumor’s location in the brain stem, surgical removal is often impossible. For radiotherapy, the dose constraint of the brain stem impedes dose escalation. Almost all patients expire within 1 year of diagnosis despite radical treatment. Boron neutron capture therapy (BNCT), a targeted radiotherapy, may have the potential to deliver an adequate radiation dose to the tumor cells while sparing the normal brain stem.

**Fig 1:**
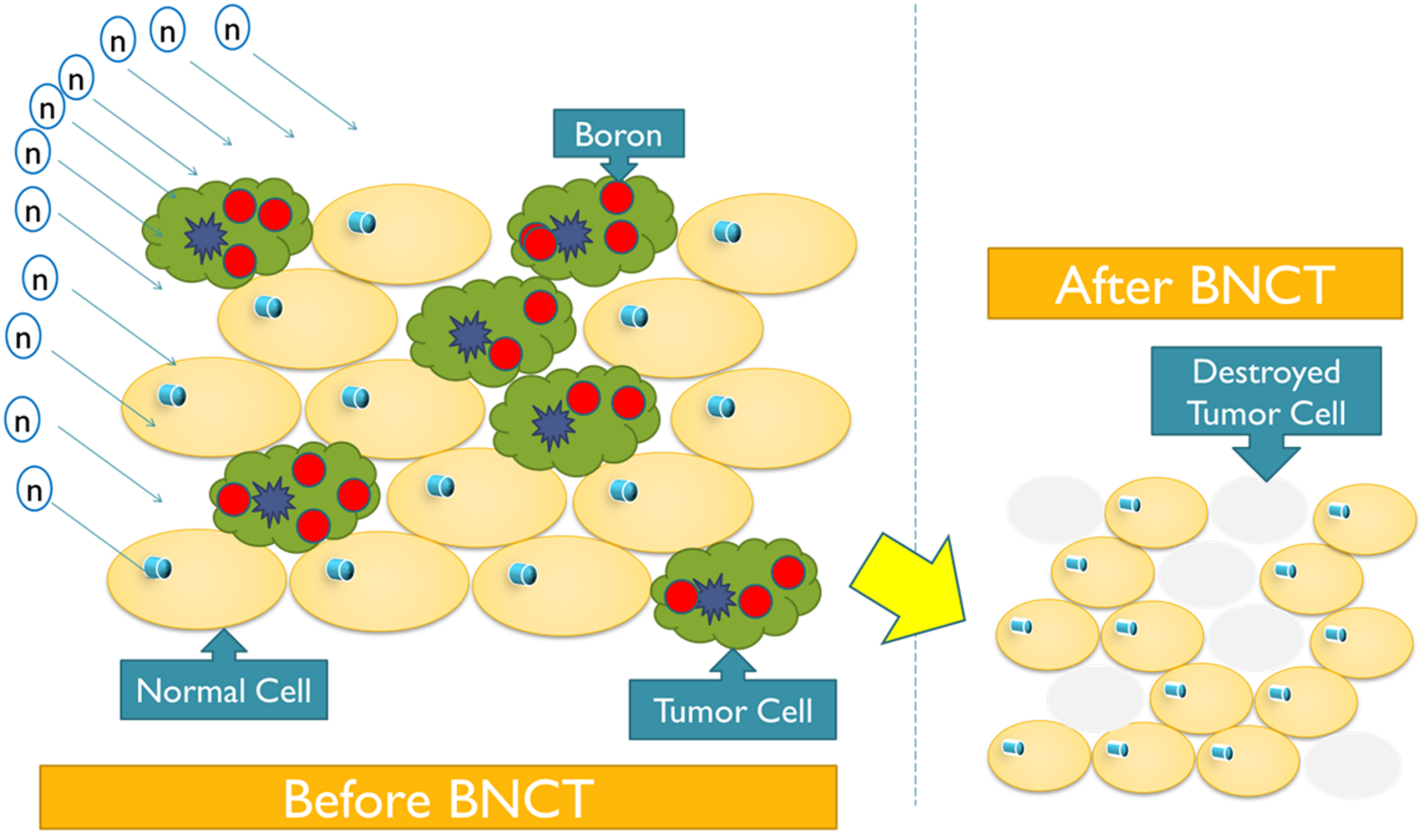
BNCT, as a targeted radiotherapy, may selectively damage cancer cells while sparing normal ones.

BNCT applies thermal neutrons to tumors that take up ^10^B selectively. Currently, there are two ^10^B-containing drugs being used in human clinical trials for BNCT: BSH (disodium mercaptoundecahydrododecaborate) and BPA (*p*-Boron-L-phenylalanine). Tumors tend to take up these drugs preferentially, such that a higher ^10^B concentration can be achieved in tumors than in normal tissue. The tumor/normal ratio (T/N ratio) is the ratio of the concentration of the ^10^B-containing drug taken up by the tumor relative to that of normal tissue. The neutron-capture reaction (^10^B(n,α)^7^Li) produces two high linear energy transfer (LET) particles with approximately a 10-μm range that damage tumor cells while substantially sparing surrounding normal tissues (Fig 1). With a high T/N ratio, targeted radiotherapy is thus made possible.

**Fig 2:**
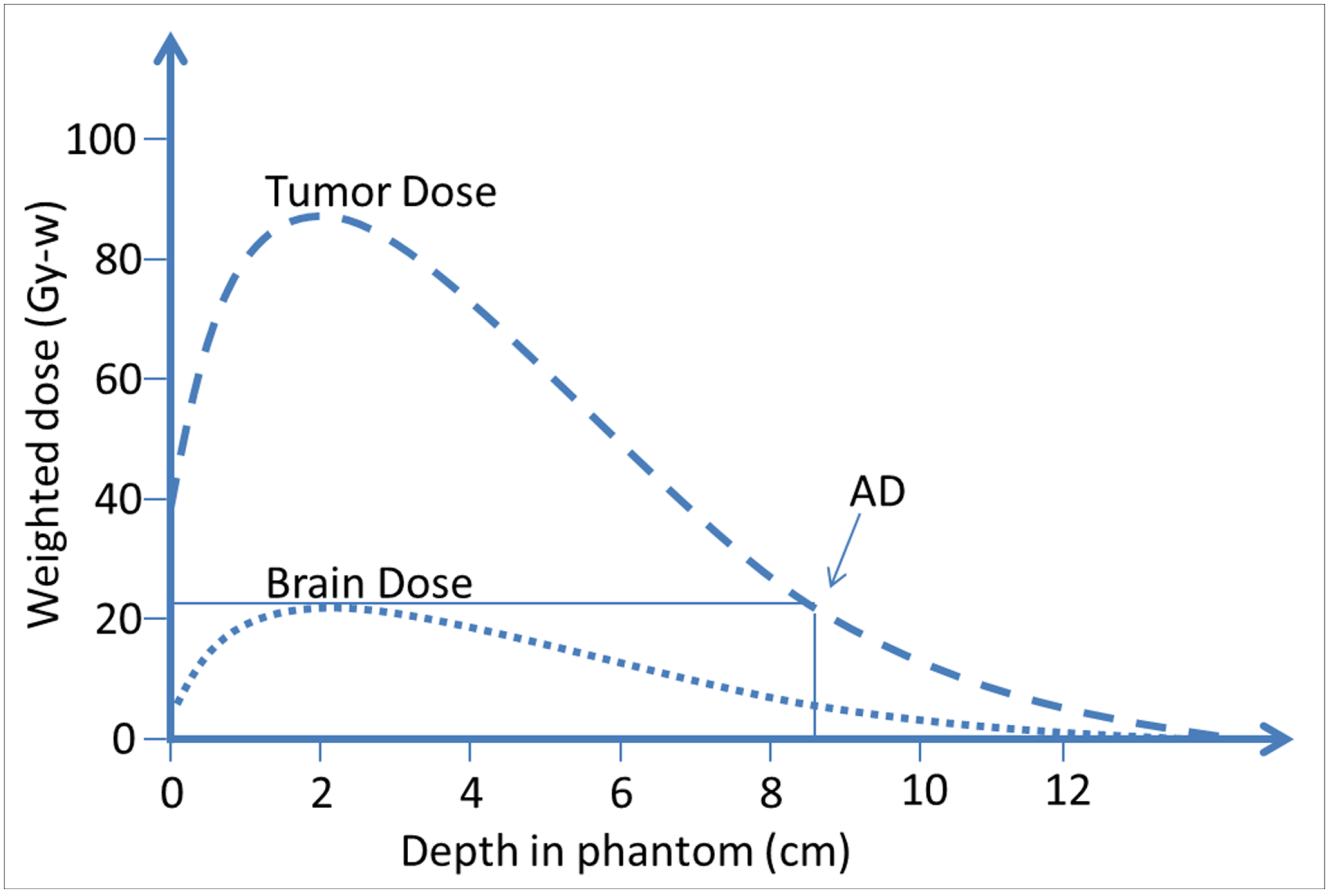
Advantage depth. The advantage depth is the depth in the tissue at which the dose to the tumor equals the maximum dose to the normal tissue along the beam. The AD of THOR is 8.5 cm, which is deeper than the deepest depth of tumor tissue, providing therapeutic benefit.

BNCT use has been limited by the advantage depth (AD), at which the dose to the tumor is the same as the maximal dose to the normal tissue on the path of the neutron beam (Fig 2). Using a 3.5 of ratio of BPA boron concentrations in tumor and normal tissue, it was found that AD was 8.5 cm. Tumor tissue deeper than the AD would be without therapeutic benefit. Attempts to treat DIPG with BNCT have been made in Japan [1], but all 6 patients treated in that study expired. The failure in that study may be partially attributed to the invasiveness of the treatment at that time and to the uncertainty of the true T/N ratio, making accurate dose estimation difficult. Since the neutron source used in that study was a thermal neutron, which has lower energy than the current standard epithermal neutron, the AD was thus inadequate without craniotomy. The patients in that study were treated intra-operatively during craniotomy. Moreover, positron emission tomography (PET) was not performed to estimate the boron uptake for dosimetric estimation, which might have led to less accurate estimations than does the current standard of performing PET as a part of dose calculation.

Efforts to increase the energy of the neutron source have been made. Neutron sources with higher energy, such as epithermal neutrons, have been used to perform BNCT as far back as 1994 [2]. With better penetration, thus a deeper AD, it might be possible to treat DIPG with BNCT without craniotomy. However, to the best of our knowledge, there has been no published study treating DIPG with BNCT in the era of epithermal neutron sources.

In this study, we aimed to perform a preliminary dosimetric investigation on the feasibility of BNCT without craniotomy in patients with DIPG and to compare the benefits of dose distribution by increasing the field number from two to four.

## Materials and methods

### Neutron source

An epithermal neutron test beam of the Tsing-Hua open-pool reactor (THOR) was constructed in 1998 for studying BNCT. A rebuilt epithermal beam port for BNCT at THOR was finished in the summer of 2004. The first clinical trial of BNCT in THOR for recurrent head and neck cancer was conducted between 2009 and 2013 [3, 4]. The AD of THOR is 8.5 cm [5].

### Patient selection

In this study, simulation computerized tomography (CT) scans of 12 consecutive patients with DIPG treated with external beam photon radiotherapy in Taipei Veterans General Hospital (TVGH), Taipei, Taiwan, from 2008 to 2015 were used for dose calculation. The patients ranged in age from 4 years to 53 years, with median age of 10 years. This study has been approved by Institutional Review Board, Taipei Veterans General Hospital (No. 2016-05-018CC). The study has been conducted according to the principles expressed in the Declaration of Helsinki. The data were analyzed anonymously.

### The treatment planning systems

We used NCTPlan Ver. 1.1.44 [6, 7], developed by the Harvard/MIT BNCT group, for treatment planning. Because NCTPlan requires CT images in tagged image file format (TIFF) files, we utilized ImageJ Ver. 1.48v [8–10] and in-house code to transform the CT images in Digital Imaging and Communications in Medicine (DICOM) to TIFF format with 256 × 256 pixels, 125 slices, and a 2-mm slice thickness. The gray level of the CT images was set to 8 bits. NCTPlan converted the input images into 21 × 21 × 25 voxels and 56 different materials, which was defined in the International Commission on Radiation Units and Measurements 46 (ICRU-46) report [11] for calculations. The doses were calculated by F4 tally and kinetic energy released in matter factors in Monte Carlo N-Particle Transport Code, Ver. 4C [12].

Dose-rate scaling factor (DRSF) is defined here as normalization factor derived individually for each dose component in a BNCT in-phantom radiation field that provides the best agreement between measured and computed data. The adjusted computed dose, D_adj_ (dose component), at any position within the ellipsoidal head phantom was then given by Eqs (1)–(4).

$${\text{D}}_{adj}\left(thermal\right)=DRSF\left(thermal\right)\times D\left(thermal\right)$$ (1)

$${\text{D}}_{adj}\left(fast\right)=DRSF\left(fast\right)\times D\left(fast\right)$$ (2)

$${\text{D}}_{adj}\left(photon\right)=DRSF\left(photon\right)\times D\left(photon\right)$$ (3)

$${\text{D}}_{adj}\left(B^{10}\right)=DRSF\left(B-10\right)\times D\left(B-10\right)$$ (4)

The dose-rate scaling factor values derived from the measured and computed depth-dose-rate distributions by least-squares criteria were 0.64, 1.39, 0.96, and 0.65, for thermal neutron, fast neutron, photon, and B-10, respectively [13]. Although the T/N ratio between boron concentrations in DIPG remains unknown, because most DIPGs are composed of glioblastoma, we assumed a T/N ratio of 3.5 according to a previous glioblastoma study [14].

Based on the results of the Monte Carlo calculations, to compute the total biologically weighted dose (RBE dose), we used the following parameters for our estimations: a relative biological effectiveness (RBE) factor of 3.8 and 1.3 for BPA in the tumor cells and brain, respectively. A RBE of 3.2 for THOR epithermal neutron beam high-LET components, such as the products of thermal neutron capture in nitrogen and fast neutrons, and a RBE of 0.5 for photon [13]. The default neutron power and flux were 1.2 MW and 1.28 × 10^9^ (n·cm^-2^·s^-1^), respectively, for the conditions of THOR [15]. RBE dose was determined by Eq (5):

$$\text{RBE dose}=RBE\left(thermal\right)\times D_{adj}\left(thermal\right)+RBE\left(fast\right)\times D_{adj}\left(fast\right)+RBE\left(photon\right)\times D_{adj}\left(photon\right)+RBE\left(B-10\right)\times D_{adj}\left(B-10\right)$$ (5)

We measured the distance from the body surface to the deepest tumor depths on CT images using the Eclipse version 13 treatment planning system (Varian Medical Systems, Palo Alto, CA, USA). Dosimetry of multiple fields was calculated from two to four fields with 14 cm-diameter collimator. The directions used in the two-field (2F) plan were left (90°) and right (270°). The directions used in the three-field (3F) plan were left anterior oblique (60°), right anterior oblique (300°), and posterior (180°). The directions used in the four-field (4F) plan were anterior (0°), left (90°), posterior (180°), and right (270°). The weightings of the multiple fields from each direction were optimized manually until the difference of the maximal dose to normal tissues of each direction was within 10%. The dose prescription was 20 Gy-Eq in 80% of the clinical target volume (CTV), as prescribed in a clinical trial of BNCT for recurrent head and neck cancer in our institution [4]. We also calculated the estimated total beam-on time for the 2F, 3F, and 4F plans.

### Delineation of target volume and normal tissue

The CTV and normal tissue were delineated by one radiation oncologist and verified by another on NCTPlan according to the MRI images that were registered with the simulation CT image on the Eclipse treatment planning system. The CTV contains the gross extent of the malignant growth and subclinical microscopic malignant disease, as defined by ICRU 62 [16].

### Dosimetric evaluation

The dose distribution in the CTV in each plan was evaluated by the following parameters: CTV minimal dose (CTV_min_), CTV maximal dose (CTV_max_), minimal dose to 95% of the CTV volume (CTV_95%_), and homogeneity index (HI). HI was applied to evaluate the homogeneity in CTV as defined by [17]:

$$HI=\frac{D_{5}}{D_{95}}$$

where D_5_ and D_95_ represent minimum dose in 5% and 95% of CTV, respectively.

The therapeutic gain, defined as the ratio of minimal dose to CTV and maximal dose to normal tissue (CTV_min_/NT_max_), was calculated. The integral dose (ID) of radiation delivered to the normal tissue was defined as $ID(Gy-Eq\cdot L)=\overset{¯}{D}(Gy-Eq)\cdot V(L)$, where $\overset{¯}{D}(Gy-Eq)$ is the mean dose delivered to volume *V*(*L*,*Liter*)as noted by Aoyama et al. [18]. Avoidance to organs at risk, including the normal brain (NB), optic nerve (ON), and circle of Willis (CW), was evaluated by the following parameters: mean dose (D_mean_), maximum dose (D_max_), and ID of the NB, ON, and CW, respectively.

### Statistical analysis

All statistical tests were performed using SPSS software (release 17.0, SPSS Inc., Chicago, IL, USA). One-way analysis of variance was used to compare dosimetric differences of the 2F, 3F, and 4F BNCT plans. Tests were two-sided. The differences were considered statistically significant if P ≤ 0.05.

## Results

### Maximal tumor depths and dose distribution

**Fig 3:**
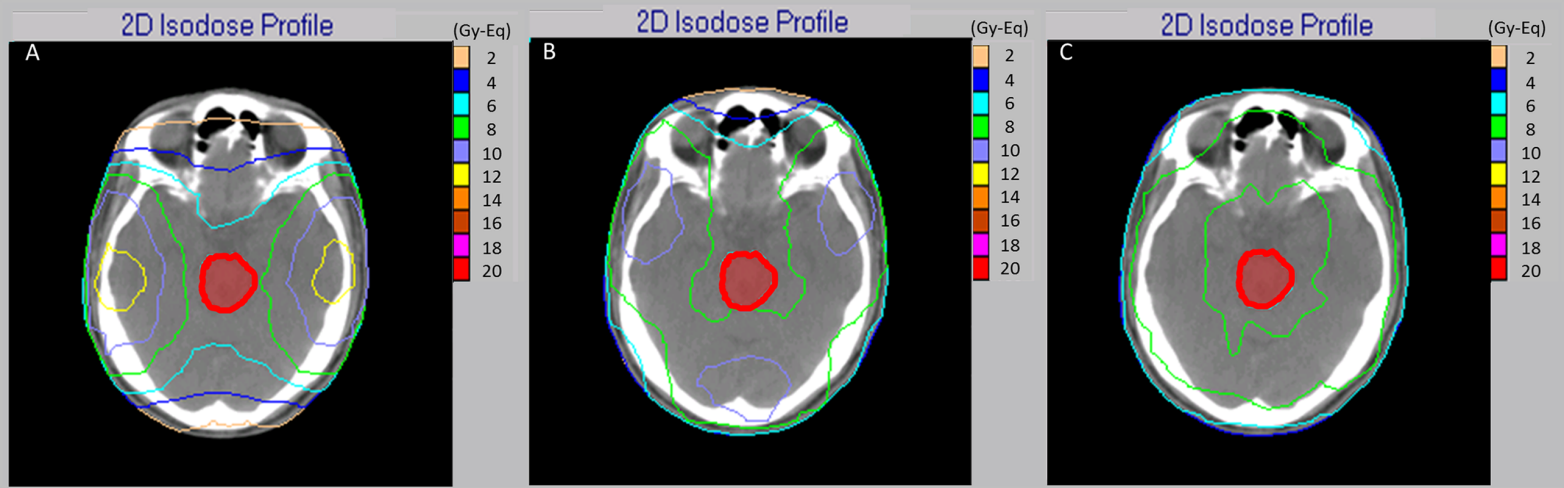
Dose distribution of 2F (a), 3F (b) and 4F (c) irradiation. A lower dose to normal brain may be obtained with 4F.

**Fig 4:**
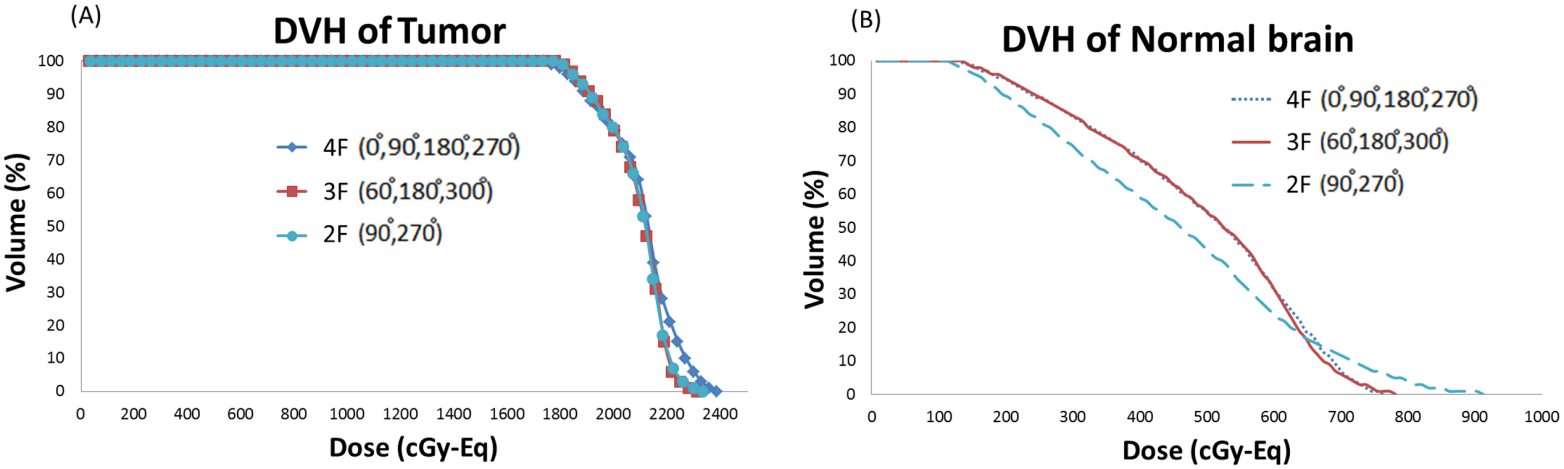
Dose volume histograms (DVHs) of 2F (a), 3F (b) and 4F (c) irradiation for one representative case.

The maximal depth of tumors from body surfaces based on CT images with 0°, 60°, 90°, 180°, 270°, and 300° beam angles are shown in Table 1. This table shows that the mean maximal tumor depths in six different beam directions were larger than the AD. Use of a single direction for DIPG is without therapeutic benefit. The dose distributions and dose volume histogram of 2F, 3F, and 4F irradiation are shown in Figs 3 and 4. With 4F irradiation, the dose affecting normal tissue can be reduced.

**Table 1:** Maximal tumor depths in each beam angle.

Beam angle	0°	60°	90°	180°	270°	300°
Mean deepest tumor depth (cm)	11.76 ± 0.65	9.77 ± 0.80	9.52 ± 0.74	9.19 ± 0.62	9.60 ± 0.80	10.04 ± 0.68
Depth range (cm)	10.79–12.78	8.53–10.85	8.3–10.31	8.52–10.18	8.56–10.76	9.11–11.00

### Target dosimetric evaluation

The minimal, maximal, and mean dose to CTV and CTV_95%_ are shown in Table 2.

**Table 2:** Patient number, age, and CTV dosimetric parameter of 2F, 3F, and 4F plans.

Patient number	Age, years	CTV_min_ (Gy-Eq)			CTV_max_ (Gy-Eq)			CTV_mean_ (Gy-Eq)			CTV_95%_ (Gy-Eq)		
		2F	3F	4F	2F	3F	4F	2F	3F	4F	2F	3F	4F
1	32	17.42	18.42	17.96	24.84	24.13	23.13	21.05	21.13	21.08	18.71	19.20	18.95
2	13	16.71	17.03	16.91	27.72	23.90	23.85	21.36	21.01	21.22	18.35	18.22	18.03
3	6	17.64	17.45	17.34	24.42	23.36	23.74	20.66	20.74	21.05	18.99	18.85	18.80
4	53	17.30	17.66	17.05	25.49	25.89	24.12	20.67	21.09	21.02	19.11	18.82	18.65
5	38	17.36	17.36	17.81	26.04	26.04	23.02	20.94	21.32	20.92	19.09	19.02	18.90
6	8	18.51	18.07	18.32	23.37	23.74	22.96	20.70	20.91	20.84	19.42	19.16	18.94
7	8	16.87	17.36	17.31	27.07	25.14	24.72	21.47	21.20	21.61	18.82	18.86	18.82
8	5	17.85	17.53	17.99	24.99	24.49	23.65	20.85	21.13	21.01	18.93	18.93	19.01
9	4	17.55	17.32	18.74	24.63	23.98	25.34	21.04	21.26	22.69	18.77	18.65	20.07
10	12	16.64	17.22	17.45	25.67	28.94	26.18	20.6	21.42	21.47	19.01	18.95	18.89
11	4	16.50	16.94	15.89	24.31	23.52	22.63	21.16	21.19	19.96	18.24	18.47	18.30
12	13	15.62	17.26	17.11	17.26	25.47	24.63	21.54	21.47	21.64	18.00	18.18	18.41
Mean	16.30	17.16	17.47	17.49	24.65	24.88	24.00	21.00	21.16	21.21	18.79	18.78	18.81
SD	15.90	0.75	0.42	0.75	2.62	1.57	1.06	0.33	0.21	0.64	0.41	0.33	0.50
P-value		2F vs. 3F (P = 0.532)			2F vs. 3F (P = 0.955)			2F vs. 3F (P = 0.690)			2F vs. 3F (P = 0.998)		
		2F vs. 4F (P = 0.485)			2F vs. 4F (P = 0.696)			2F vs. 4F (P = 0.512)			2F vs. 4F (P = 0.987)		
		3F vs. 4F (P = 0.997)			3F vs. 4F (P = 0.516)			3F vs. 4F (P = 0.955)			3F vs. 4F (P = 0.975)		

There was no significant difference in CTV_min_, CTV_max_, CTV_mean_, and CTV_95%_ among the three settings. CTV_min_/NT_max_, HI, and beam-on time are shown in Table 3.

**Table 3:** CTV_min_/NT_max_, HI, and beam-on time of 2F, 3F and 4F plans.

Patient number	CTV_min_/NT_max_			HI			Beam-on time (min)		
	2F (A)	3F (B)	4F (C)	2F (A)	3F (B)	4F (C)	2F (A)	3F (B)	4F (C)
1	1.97	2.51	2.77	1.18	1.23	1.21	45.40	48.90	49.60
2	1.48	2.05	2.24	1.31	1.26	1.28	55.20	54.60	57.60
3	1.75	2.16	2.40	1.18	1.23	1.21	45.00	50.40	52.40
4	1.4	1.64	1.92	1.19	1.27	1.24	60.20	74.70	73.20
5	1.45	1.92	2.36	1.21	1.29	1.19	59.00	60.90	59.20
6	2.23	2.42	2.71	1.14	1.20	1.17	40.80	49.20	49.20
7	1.55	2.03	2.32	1.29	1.25	1.25	52.00	51.30	56.00
8	1.82	2.24	2.62	1.23	1.25	1.20	46.80	49.20	51.60
9	2.04	2.36	2.64	1.23	1.26	1.22	40.20	46.20	50.80
10	1.25	1.81	2.06	1.28	1.35	1.30	63.80	62.40	66.00
11	2.05	2.45	2.43	1.29	1.23	1.24	37.60	40.50	42.00
12	1.68	1.92	2.40	1.34	1.36	1.32	47.80	57.90	53.20
Mean	1.72	2.13	2.41	1.24	1.27	1.24	49.48	53.85	55.07
SD	0.31	0.28	0.26	0.06	0.05	0.05	8.52	9.03	8.25
P-value	2F vs. 3F (P = 0.005)*			2F vs. 3F (P = 0.489)			2F vs. 3F (P = 0.470)		
	2F vs. 4F (P < 0.001)*			2F vs. 4F (P = 0.988)			2F vs. 4F (P = 0.296)		
	3F vs. 4F (P = 0.064)			3F vs. 4F (P = 0.404)			3F vs. 4F (P = 0.942)		

The CTV_min_/NT_max_ of the 4F (P < 0.001) and 3F (P = 0.005) were better than that of 2F. CTV_min_/NT_max_ of 4F was slightly larger than that of 3F (P = 0.064). HI and beam-on time were not significantly different among the three plans.

### Radiation exposure of organs at risk

The volume, D_mean_, D_max_, and ID of the NB, ON, and CW for 2F, 3F, and 4F plans are listed in Table 4.

**Table 4:** Volume, D_mean_, D_max_, ID of NB, ON, CW and beam on time for 2F, 3F, and 4F plans.

Organ	Volume (cm^3^)	Parameter	2F	3F	4F	P-value	Tolerance reference
NB	1166.21 ± 121.20	D_mean_ ^a^	4.46 ± 0.40	4.66 ± 0.42	4.56 ± 0.35	2F vs. 3F (P = 0.801)	Max. dose < 13 Gy-Eq [19]
						2F vs. 4F (P = 0.862)	
						3F vs. 4F (P = 0.486)	
		D_max_ ^a^	10.22 ± 1.72	8.34 ±1.10	7.35 ± 0.72	2F vs. 3F (P = 0.160)	
						2F vs. 4F (P < 0.001)*	
						3F vs. 4F (P = 0.004)*	
		ID ^b^	523.77 ± 93.89	547.03 ± 101.76	534.68 ± 94.46	2F vs. 3F (P = 0.842)	
						2F vs. 4F (P = 0.966)	
						3F vs. 4F (P = 0.948)	
L-ON	1.72 ± 0.55	D_mean_	4.23 ± 0.70	5.60 ± 0.57	6.09 ± 0.44	2F vs. 3F (P = 0.139)	Max. dose < 10 Gy[20]
						2F vs. 4F (P < 0.001)*	
						3F vs. 4F (P < 0.001)*	
		D_max_	5.09 ± 0.51	6.07 ± 0.68	6.60 ± 0.44	2F vs. 3F (P = 0.087)	
						2F vs. 4F (P < 0.001)*	
						3F vs. 4F (P = 0.001)*	
		ID	0.69 ± 0.19	0.96 ± 0.32	1.04 ± 0.31	2F vs. 3F (P = 0.077)	
						2F vs. 4F (P = 0.018)*	
						3F vs. 4F (P = 0.801)	
R-ON	1.59 ± 0.38	D_mean_	4.23 ± 0.50	5.59 ± 0.55	6.19 ± 0.55	2F vs. 3F (P = 0.038)*	Max. dose < 10 Gy[20]
						2F vs. 4F (P < 0.001)*	
						3F vs. 4F (P < 0.001)*	
		D_max_	5.24 ± 0.48	6.11 ± 0.64	6.65 ± 0.51	2F vs. 3F (P = 0.077)	
						2F vs. 4F (P < 0.001)*	
						3F vs. 4F (P = 0.002)*	
		ID	0.66 ± 0.14	0.89 ± 0.24	0.98 ± 0.24	2F vs. 3F (P = 0.038)*	
						2F vs. 4F (P = 0.003)*	
						3F vs. 4F (P = 0.580)	
CW	1.00 ± 0.22	D_mean_	4.79 ± 0.36	5.26 ± 0.40	5.59 ± 0.31	2F vs. 3F (P = 0.096)	Max. dose <12 Gy-Eq [1]
						2F vs. 4F (P < 0.001)*	
						3F vs. 4F (P = 0.012)*	
		D_max_	5.35 ± 0.38	5.60 ± 0.43	5.99 ± 0.37	2F vs. 3F (P = 0.063)	
						2F vs. 4F (P = 0.001)*	
						3F vs. 4F (P = 0.314)	
		ID	0.48 ± 0.10	0.48 ± 0.18	0.51 ± 0.20	2F vs. 3F (P = 0.999)	
						2F vs. 4F (P = 0.903)	
						3F vs. 4F (P = 0.898)	

NB: normal brain, L-ON: left optic nerve, R-ON: right optic nerve, CW: circle of Willis, D_mean_: mean dose, D_max_: maximum dose, ID: integral dose.

^a^ in Gy-Eq.

^b^ in cGy-Eq-L

There was no significantly difference in D_mean_ and ID of NB among the three plans. D_max_ to NB was lowered with the 4F plan.

D_mean_ and D_max_ to optic nerves were higher by 4F. Compared with the 2F plan, 4F yielded significant increases in ID to the optic nerves.

There was no significant difference in ID to CW among the three settings. The D_max_ to CW by the 4F plan was higher than that of the 2F plan. The dose constraints may be fulfilled by all three settings.

## Discussion

In this study, we reviewed the simulation CT scans of 12 consecutive patients treated by conventional radiotherapy in our institution. We found that, without craniotomy, the prescribed therapeutic dose to the CTV may be fulfilled while complying with the dose constraints to the NB, ON, and CW. Specifically, a therapeutic benefit may be achieved by applying multi-field BNCT to treat DIPG without craniotomy, while the therapeutic gain, defined by CTV_min_/NT_max_, was greatest in 3F and 4F plans. To the best of our knowledge, the current study is the first to show that, dosimetrically, treatment of DIPG by BNCT without craniotomy may be feasible.

Besides the normal tissue constraints referred to in Table 4, Harvard-MIT and the Brookhaven National Laboratory Phase I and Phase I/II clinical trials found that the doses associated with a 50% incidence for somnolence (ED_50_ ± SE) were 6.2 ± 1.0 Gy-Eq for the average whole-brain dose and 14.1 ± 1.8 Gy-Eq for the peak brain dose [21]. In the present study, we found that when multi-field BNCT was applied, the mean and maximal doses to the brain were below the aforementioned ED50 levels.

The Harvard-MIT trial data also suggested that the increased number of radiation fields may be the major reason for increased average whole-brain dose. However, in the present study, we found that when we applied a four-field plan, the average whole-brain dose *decreased*. The reason for this discrepancy is likely due to the fact that that in the previous trial, the clinicians increased radiation fields for dose escalation. Instead, in the present study, the prescribed dose remained the same, while the number of radiation fields was increased.

For a disease of involving predominantly children, the duration of treatment is crucial. In this study, we found that the average beam-on time was within 1 hour, which should be feasible for treatment of pediatric patients.

The outcome of BNCT in treating glioblastoma has been shown to be comparable with conventional radiotherapy with concurrent temozolomide, with a much shorter treatment duration [22]. The present findings suggest that treatment of DIPGs with multi-beam BNCT may have a therapeutic benefit dosimetrically. Given that most DIPGs are glioblastomas, a similar outcome may be expected. A clinical trial is warranted for this grave disease.

The actual T/N ratio of DIPG is unknown. The normal tissue tolerance of pediatric patients may be different from that of adults. To further reduce the dose to normal tissues, blockers should be applied in future studies.

Although the beam-on time in two, three, and four port irradiation was almost the same, if we consider the interval time for set-up of the patient, the treatment time in four-port irradiation may be much longer compared with two-port irradiation.

## Conclusions

Therapeutic benefits may be achieved using multi-field BNCT in patients with DIPG. The maximal dose to normal brain would be minimal and the prescription dose to tumor would be higher by four fields. To initiate a clinical trial on this devastating disease might be worthwhile.
